# Umbrella review of anticoagulation strategies for secondary prevention of cardioembolic stroke in atrial fibrillation: efficacy, safety, and knowledge gaps

**DOI:** 10.1097/MS9.0000000000004056

**Published:** 2025-10-15

**Authors:** Nicholas Aderinto, Israel Charles Abraham, Ahmedyar Hasan, Abieyuwa Tari-Ere Oshodin, Obianuju Iheomamere Muoghallu, Ebube Christopher Mbah, Florence Oluwatoyin Akintepede, Aderinola Halimat Abioye, Sulaiman Olaide Bukky, Morounfoluwa P. Olalusi, Emmanuel Kokori, Gbolahan Olatunji

**Affiliations:** a Department of Medicine, LAUTECH Teaching Hospital, Ogbomoso, Nigeria; b Department of Medicine and Surgery, University of Ilorin, Ilorin, Nigeria; c Neurology Department, University of Minnesota, Minneapolis, MN, USA; d Department of Medicine and Surgery, Obafemi Awolowo University Teaching Hospital, Ife, Nigeria; e Department of Medicine, University of Uyo Teaching Hospital, Uyo, Nigeria; f Department of Public Health, Ahmadu Bello University, Zaria, Nigeria; g Department of Medicine, Ladoke Akintola University Teaching Hospital, Ogbomoso, Nigeria; h Department of Internal Medicine, Alpha Specialist Hospital, Ibadan, Nigeria; i Emergency Medicine Department, Accident and Emergency, Mid Cheshire NHS Trust, Cheshire, UK; j Department of Medicine, AMA School of Medicine, Makati, Philippines

**Keywords:** anticoagulation, atrial fibrillation, cardioembolic stroke, direct oral anticoagulants (DOACs), secondary prevention, vitamin K antagonists (VKAs)

## Abstract

Atrial fibrillation (AF) is a major contributor to cardioembolic stroke. Anticoagulation, guided by CHA2DS2-VASc and HAS-BLED scores, is critical for secondary prevention, yet challenges persist in timing, patient-specific tailoring, and balancing efficacy with bleeding risk. This umbrella review synthesizes evidence on anticoagulation strategies for secondary prevention of cardioembolic stroke in AF patients. We searched PubMed, Scopus, Cochrane Library, Web of Science, and Embase for systematic reviews and meta-analyses (inception to May 2025) assessing oral anticoagulants (OACs) [vitamin K antagonists (VKAs), direct oral anticoagulants (DOACs)] in AF patients with prior stroke or transient ischemic attack (TIA). Outcomes included recurrent stroke, systemic embolism, major bleeding, intracranial hemorrhage (ICH), and mortality. Quality was assessed using AMSTAR-2. Data were narratively synthesized, with quantitative metrics extracted where feasible. Eight reviews (2007–2025) were included, comprising 4–52 studies and 6893–94 656 participants. DOACs demonstrated superior efficacy over VKAs [apixaban odds ratio (OR) 0.79, 95% confidence interval (CI) 0.66–0.94], and VKAs outperformed antiplatelets (relative risk reduction 64%, absolute risk reduction 8.4%/year). DOACs showed lower major bleeding and ICH rates than VKAs, with apixaban exhibiting the most favorable safety profile (OR 0.71, 95% CI 0.61–0.81). Early versus delayed OAC initiation post-stroke improved efficacy (OR 0.68, 95% CI 0.55–0.84) with comparable safety. Identified knowledge gaps include optimal timing of OACs, outcomes in high-risk subgroups (elderly, renal impairment, and post-ICH), and cost-effectiveness. DOACs are superior to VKAs for secondary prevention of cardioembolic stroke in AF. However, individualized approaches are needed for timing and high-risk subgroups.

## Introduction

Atrial fibrillation (AF) is the most common cause of cardioembolic stroke, which represents a more severe subtype of ischemic stroke with a higher risk of recurrence^[1,2]^. Cardioembolic strokes account for approximately 20–25% of all ischemic strokes and are associated with greater morbidity and mortality than other stroke subtypes^[2]^. The risk of cardioembolic stroke in patients with AF is typically assessed using the CHA_2_DS_2_-VASc score, which categorizes patients as low (score 0), moderate/intermediate (score 1–2), or high risk (score ≥3)^[3]^. AF increases the risk of ischemic stroke by approximately three- to five-fold and is estimated to contribute to 15% of strokes globally^[4]^.

The prevention of cardioembolic stroke in AF relies on evaluating both stroke and bleeding risk using the CHA_2_DS_2_-VASc and HAS-BLED scores, respectively, followed by the appropriate initiation of oral anticoagulant (OAC) therapy^[5]^. However, challenges persist in clinical settings. The optimal timing for initiating OAC, particularly after a severe stroke, remains uncertain. Moreover, the cost-effectiveness of direct oral anticoagulants (DOACs) remains a concern, especially in low-resource settings. Standardized stroke prevention protocols can lead to under- or overtreatment due to variations in patient risk profiles, comorbidities, affordability, and prior medical history^[6]^. These issues show the need for individualized treatment strategies guided by stroke severity, imaging findings, risk scores, and the selection of appropriate OAC.

Historically, vitamin K antagonists (VKAs), such as warfarin, have been the cornerstone of pharmacologic stroke prevention in AF since the 1950s, though both have notable limitations^[6]^. The advent of DOACs has marked a significant advancement, offering improved efficacy, a lower risk of bleeding, fixed dosing, and eliminating the need for regular international normalized ratio (INR) monitoring^[6]^. Current guidelines from the American Heart Association/American Stroke Association (AHA/ASA) recommend DOACs over VKAs as first-line therapy for patients with AF, except in cases of moderate to severe valvular heart disease (e.g., mitral stenosis) or in recipients of mechanical heart valves^[6]^.

While several systematic reviews have assessed the efficacy and safety of OACs’ overall efficacy and safety, most studies have combined data on both primary and secondary prevention. There remains a lack of focused evidence specifically addressing the secondary prevention of cardioembolic stroke in patients with AF. This gap is particularly relevant for high-risk subgroups, such as older adults, patients with a recent acute coronary syndrome (ACS), renal or hepatic impairment, and women.

This umbrella review aims to synthesize evidence from existing systematic reviews and meta-analyses to evaluate the efficacy and safety of OAC strategies for the secondary prevention of cardioembolic stroke in patients with AF. This paper complies with the TITAN guidelines on AI reporting^[7]^.

## Methodology

### Study design

This umbrella review synthesizes and maps evidence from existing systematic reviews and meta-analyses on anticoagulation strategies for the secondary prevention of cardioembolic stroke in patients with AF, evaluates their efficacy and safety, and identifies knowledge gaps.

### Eligibility criteria

- **Population**: Adult patients diagnosed with AF and a history of stroke or transient ischemic attack (TIA). Reviews that combined mixed populations (primary and secondary prevention) were retained if they included a proportion of prior stroke/TIA patients. These are reported as indirect evidence and interpreted with caution.
- **Intervention**: OACs, including DOACs (e.g., apixaban, dabigatran, edoxaban, and rivaroxaban), warfarin, and combination therapies.
- **Comparison**: Alternative anticoagulation therapies, antiplatelet agents (e.g., aspirin), or placebo.
- **Outcomes**: Primary outcomes included recurrent stroke (ischemic or cardioembolic) and systemic embolism. Secondary outcomes encompassed major bleeding, intracranial hemorrhage (ICH), mortality, and functional outcomes.
- **Inclusion criteria**: Systematic reviews and meta-analyses (head-to-head or network analyses) published in English from database inception to 15 May 2025.
- **Exclusion criteria**: Studies focused exclusively on primary prevention, non-AF-related stroke, non-English studies, or non-systematic reviews (e.g., narrative reviews and individual trials).

**HIGHLIGHTS**

- Direct oral anticoagulants (DOACs) outperform warfarin: DOACs (e.g., apixabanand dabigatran) reduce recurrent stroke/systemic embolism more effectively than warfarin [apixaban odds ratio (OR) 0.79, 95% confidence interval (CI) 0.66–0.94] with lower bleeding/intracranial hemorrhage (ICH) risk (apixaban OR 0.71, 95% CI 0.61–0.81).
- Early anticoagulation effective: Starting anticoagulation ≤14 days post-stroke reduces recurrent stroke by 32% (OR 0.68, 95% CI 0.55–0.84) without increased bleeding risk [relative risk (RR) 1.10, 95% CI 0.61–1.99].
- Gaps in high-risk groups: limited evidence exists for optimal anticoagulation in elderly, renal-impaired, or post-ICH patients, requiring further research on timing and tailored approaches.

### Information sources

- **Databases**: PubMed, Scopus, Cochrane Library, Web of Science, Google Scholar, and Embase.

### Search strategy and study selection

**Figure 1.**
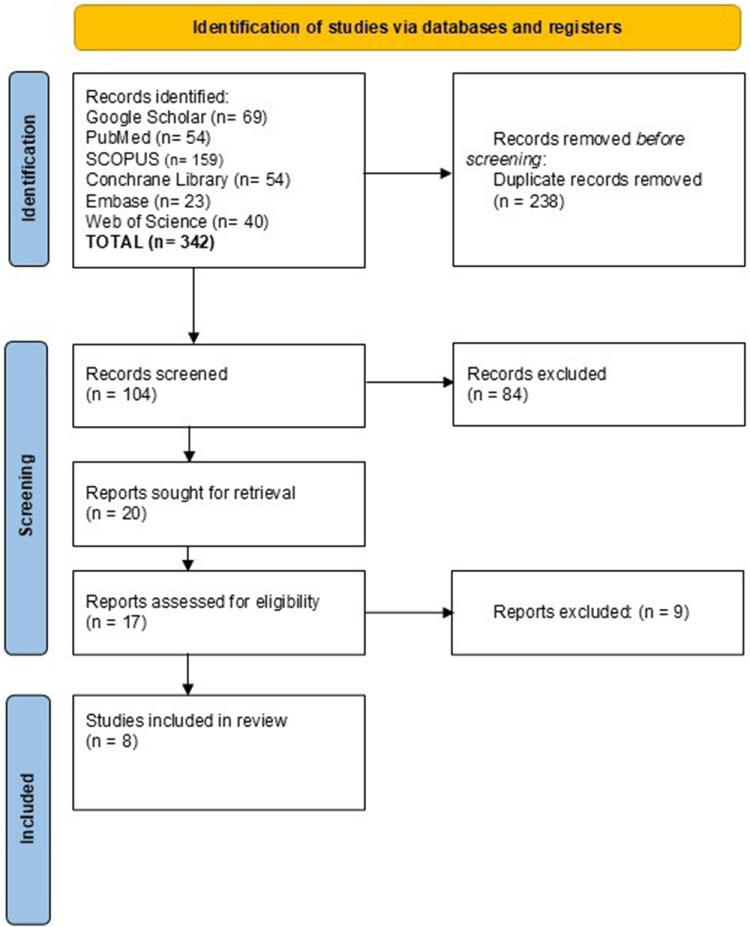
PRISMA flowchart for study selection.

A search strategy was developed with two independent reviewers (N.A. and I.C.A.) to maximize sensitivity and specificity. (“Atrial Fibrillation”[MeSH Terms] OR “nonvalvular atrial fibrillation”[tiab] OR “AF”[tiab] OR “auricular fibrillation”[tiab]) AND (“Anticoagulants”[MeSH Terms] OR “anticoagulants”[tiab] OR “direct oral anticoagulants”[tiab] OR “DOACs”[tiab] OR “warfarin”[tiab] OR “vitamin K antagonists”[tiab] OR “apixaban”[tiab] OR “dabigatran”[tiab] OR “edoxaban”[tiab] OR “rivaroxaban”[tiab] OR “antiplatelet agents”[MeSH Terms] OR “antiplatelet”[tiab] OR “aspirin”[tiab]) AND (“secondary prevention”[MeSH Terms] OR “secondary prevention”[tiab] OR “stroke prevention”[tiab] OR “recurrent stroke”[tiab] OR “cardioembolic stroke”[tiab] OR “ischemic stroke”[tiab] OR “transient ischemic attack”[tiab] OR “TIA”[tiab]) AND (“systematic review”[publication type] OR “Meta-Analysis”[Publication Type] OR “systematic review”[tiab] OR “meta-analysis”[tiab] OR “meta-analyses”[tiab]) AND Language: English. The search was adapted for each database (Supplemental Digital Content File S1, available at: http://links.lww.com/MS9/A1000. Two reviewers independently screened titles, abstracts, and full texts using Covidence software. Discrepancies were resolved through discussion or consultation with a third reviewer. The selection process is documented in a PRISMA flowchart (Fig. 1).

### Data extraction

- **Process**: Two reviewers independently extracted data using a standardized Excel template. A third reviewer resolved discrepancies through discussion or adjudication.
- **Data Items:**

- **Study characteristics**: Author, publication year, country, study design [e.g., randomized controlled trials (RCTs) and non-randomized studies (NR)], sample size.
- **Population**: Age, sex, and comorbidities (e.g., hypertension and diabetes).
- **Interventions**: Type, dose, and duration of anticoagulation (e.g., DOACs, warfarin, and combination therapies).
- **Outcomes**: Efficacy (recurrent stroke and systemic embolism) and safety (major bleeding, ICH, mortality, and functional outcomes).
- **Quality**: Methodological quality assessment results.

### Quality assessment

The AMSTAR-2 (a measurement tool to assess systematic reviews) tool assessed the methodological quality of included systematic reviews and meta-analyses^[8]^. Studies were rated as high, moderate, low, or critically low quality based on search comprehensiveness, risk of bias assessment, and heterogeneity analysis. Two reviewers independently conducted the evaluation, with discrepancies resolved by consensus (Table 1).

**Table 1:** AMSTAR-2 quality assessment of included reviews

Study	Year	Comprehensive search	Risk of bias assessed	Heterogeneity evaluated	AMSTAR-2 rating
Hart *et al*	2007	Yes	Yes	Yes	High
Lin *et al*	2015	Yes	Yes	Yes	High
López-López *et al*	2017	Yes	Yes	Yes	High
Almutairi *et al*	2017	Yes	Yes	Partial	Moderate
Murthy *et al*	2017	Yes	Partial	Yes	Moderate
Zhang *et al*	2021	Yes	Yes	Yes	High
Jiang *et al*	2023	Yes	Yes	Yes	High
Ntaios *et al*	2025	Yes	Yes	Yes	High

### Data synthesis

- **Approach**: A narrative synthesis was performed, structured around key themes:

- **Anticoagulation type**: Efficacy and safety of DOACs, warfarin, and combination therapies.
- **Timing**: Early versus delayed initiation of anticoagulation post-stroke.
- **Subgroups**: Analysis by DOAC type, stroke severity, and patient characteristics (e.g., age, sex, and comorbidities).

## Results

### Study characteristics

Eight systematic reviews and meta-analyses^[9–16]^, published between 2007 and 2025, were included in this umbrella review. These studies incorporated 4–52 primary studies, with total sample sizes ranging from 6893 to 94 656 participants. The populations primarily consisted of patients with AF, with a focus on those with prior ischemic stroke or TIA. Interventions assessed included warfarin, DOACs, antiplatelets, and combination therapies, compared against placebo, antiplatelets, or alternative anticoagulants. Primary outcomes encompassed recurrent stroke, systemic embolism, major bleeding, ICH, and mortality. Study designs varied, with five reviews including only RCTs and three incorporating both RCTs and non-randomized studies. Detailed characteristics are provided in Table 2.

**Table 2:** Characteristics of included systematic reviews and meta-analyses

Study	Year	Study type	No. of studies (patients)	Population	Prevention type	Interventions	Outcomes
Hart *et al*	2007	Meta-analysis	29 RCTs (28 044)	AF, 20% prior stroke/TIA	Mixed	VKAs and antiplatelets vs. placebo	Recurrent stroke and major bleeding
Lin *et al*	2015	Network meta-analysis	25 RCTs and 24 studies (NR)	AF, prior stroke/TIA subgroup	Secondary	DOACs, VKAs, and antiplatelets	Recurrent stroke, major bleeding, and ICH
López-López *et al*	2017	Network meta-analysis	23 RCTs (94 656)	AF, 20–30% prior stroke/TIA	Mixed	DOACs, VKAs, and antiplatelets	Recurrent stroke/systemic embolism, major bleeding, and mortality
Almutairi *et al*	2017	Meta-analysis	13 RCTs and 17 studies (NR)	AF, prior stroke/TIA subgroup	Secondary	DOACs vs. VKAs	Recurrent stroke, major bleeding, and ICH
Murthy *et al*	2017	Meta-analysis	8 studies (NR)	AF, post-ICH	Secondary	OACs (VKAs and DOACs)	Recurrent stroke and major bleeding/ICH
Zhang *et al*	2021	Meta-analysis	7 RCTs and 29 studies (NR)	AF, prior stroke/TIA subgroup	Secondary	DOACs vs. DOACs	Recurrent stroke/systemic embolism, major bleeding, and ICH
Jiang *et al*	2023	Meta-analysis	12 studies (11 421)	AF, post-ischemic stroke	Secondary	Early vs. delayed OACs	Recurrent stroke, hemorrhagic transformation, and major bleeding
Adamou *et al*	2025	Meta-analysis	4 RCTs (6893)	Ischemic stroke and AF subgroup	Secondary	OACs + antiplatelets	Recurrent stroke and major bleeding

### Quality assessment

The methodological quality of included reviews was evaluated using the AMSTAR-2 tool. Six reviews were rated as high quality, two as moderate quality, and none were rated low or critically low. The results of the quality assessment are summarized in Table 1.

### Anticoagulation versus control

A landmark meta-analysis by Hart *et al*^[9]^ synthesized five RCTs. It demonstrated that adjusted-dose warfarin reduced the risk of all stroke (ischemic and hemorrhagic) by 64% compared to placebo in patients with AF [absolute risk reduction (ARR) 8.4% per year, number needed to treat (NNT) = 12]. In contrast, aspirin yielded a modest 22% relative risk (RR) reduction, underscoring the superior efficacy of anticoagulants for secondary prevention of cardioembolic stroke in this population^[9]^. These findings are summarized in Table 3.

**Table 3:** Knowledge gaps by theme

Theme	Description	Studies noting gap
Limited Secondary prevention focus	Combined primary/secondary prevention data reduce specificity for prior stroke/TIA	Hart *et al*^[1]^, López-López *et al*^[3]^, and Zhang *et al*^[6]^
Sparse subgroup data	Insufficient data on elderly, post-ACS, or renal impairment patients	Almutairi *et al*^[4]^
Underexplored clinical questions	Unclear optimal timing of OACs, especially in severe stroke; limited cost-effectiveness, real-world, and personalized therapy data	Jiang *et al*^[7]^, López-López *et al*^[3]^, Murthy *et al*^[5]^, and Ntaios *et al*^[8]^

### DOACs versus VKAs

Recent evidence highlights the enhanced efficacy of DOACs over VKAs in reducing thromboembolic events. A network meta-analysis by López-López *et al*^[16]^, encompassing 23 RCTs, found that apixaban (OR 0.79, 95% confidence interval (CI) 0.66–0.94) and dabigatran (OR 0.65, 95% CI 0.52–0.81) outperformed warfarin in reducing the composite outcome of stroke or systemic embolism in AF patients, including those with prior stroke or TIA^[3]^. Similarly, Lin *et al*^[15]^ and Almutairi *et al*^[9]^ reported that DOACs, notably apixaban and dabigatran, were associated with lower rates of recurrent stroke in AF patients with a history of stroke or TIA^[10,12]^. Zhang *et al*^[14]^ further corroborated these findings in a meta-analysis of 12 RCTs, noting a 19% reduction in stroke or systemic embolism and a 50% reduction in hemorrhagic stroke with DOACs compared to warfarin, reinforcing their value for secondary stroke prevention^[14]^.

### Timing of anticoagulation initiation

Jiang *et al*^[12]^ examined the impact of early (≤14 days) versus delayed (>14 days) initiation of OACs in AF patients’ post-ischemic stroke. Early initiation was linked to a significant 32% relative reduction in recurrent ischemic stroke (OR 0.68, 95% CI 0.55–0.84), with no notable increase in bleeding risk, suggesting that starting anticoagulation within 4–14 days of an index stroke optimizes efficacy while maintaining acceptable safety.

### Special population: patients with prior ICH

Murthy *et al*^[10]^ conducted a meta-analysis to assess the efficacy of resuming anticoagulation in AF patients following ICH. The study found a pooled RR of 0.34 (95% CI 0.25–0.45) for thromboembolic events, including ischemic stroke, among patients who resumed anticoagulation after ICH. In the studies synthesized by Murthy *et al*, anticoagulation was typically resumed in carefully selected ICH survivors^[10]^. This would be adults with AF in whom neurological status was stable, blood pressure was controlled, and follow-up neuroimaging showed no ongoing bleeding or hematoma expansion; patients with active bleeding, a need for intensive antiplatelet therapy, or unresolved structural lesions were generally not restarted. Within these cohorts, resumption of OAC was associated with lower thromboembolic events and no clear increase in recurrent ICH (pooled RR 1.01, 95% CI 0.58–1.77).

### Combination therapy

Ntaios *et al*^[13]^ evaluated dual therapy combining OACs and antiplatelet agents in AF patients with ischemic stroke. This approach reduced the risk of recurrent ischemic events by 18% (*P* < 0.01), but was associated with a significant increase in major bleeding risk (RR 1.45, 95% CI 1.12–1.88). These results suggest that the net clinical benefit of combination therapy requires individualization based on thrombotic and hemorrhagic risk profiles.

### Major bleeding and intracranial hemorrhage

Hart *et al*^[14]^ reported an elevated risk of major bleeding with warfarin compared to placebo or aspirin in patients with AF, with an absolute increase of approximately 0.3% per year. However, this risk was outweighed by the substantial reduction in ischemic stroke, supporting warfarin’s net benefit for secondary prevention. Subsequent meta-analyses consistently demonstrated that DOACs have a safer profile than warfarin. López-López *et al*^[16]^, Almutairi *et al*^[9]^, and Zhang *et al*^[11]^ found that DOACs were associated with significantly lower risks of major bleeding and ICH compared to warfarin. Notably, apixaban exhibited the most favorable bleeding profile (OR 0.71, 95% CI 0.61–0.81 for major bleeding), followed by dabigatran and edoxaban, highlighting heterogeneity in bleeding risk across DOAC agents^[11]^.

### Safety of early anticoagulation

Jiang *et al*^[12]^ investigated the safety of early (≤14 days) versus delayed (>14 days) initiation of OACs in AF patients post-ischemic stroke. The meta-analysis revealed no statistically significant increase in major bleeding with early initiation (RR 1.10, 95% CI 0.61–1.99), supporting the safety of starting OACs within 14 days of a non-cardioembolic ischemic stroke.

### Anticoagulation after ICH

Murthy *et al*^[10]^ examined the safety of resuming anticoagulation in AF patients following ICH. The study found no significant increase in the risk of recurrent hemorrhage [adjusted hazard ratio (aHR) 1.01, 95% CI 0.58–1.77] among those who restarted OACs, suggesting that anticoagulation can be safely resumed in carefully selected patients with prior ICH and high ischemic risk.

### Combination therapy and bleeding risk

Ntaios *et al*^[13]^ assessed the safety of combining OACs with antiplatelet agents in AF patients with ischemic stroke. The pooled analysis reported a 45% higher RR of major bleeding, including gastrointestinal and intracranial hemorrhage, with dual therapy compared to OAC monotherapy (RR 1.45, 95% CI 1.12–1.88). This finding underscores the need for cautious consideration of indications, duration, and patient-specific risk profile before initiating combination regimens.

## Discussion

This umbrella review synthesized evidence from eight meta-analyses evaluating the efficacy and safety of various anticoagulation strategies for secondary prevention of cardioembolic stroke in patients with AF. Six of the included meta-analyses were rated as high quality based on AMSTAR-2 criteria. The collective evidence supports the superiority of DOACs over VKAs, particularly warfarin, for reducing the risk of stroke recurrence (high-certainty evidence).

Additionally, warfarin was found to be more effective than aspirin. DOACs were associated with a lower risk of major bleeding compared to warfarin. However, this benefit was not consistently observed for agents such as rivaroxaban and high-dose dabigatran.Additionally, warfarin was found to be more effective than aspirin for preventing recurrent stroke in AF patients^[14]^. DOACs were associated with a lower risk of major bleeding compared to warfarin^[17]^, although this benefit was not consistently observed for rivaroxaban or high-dose dabigatran^[17,18]^.

Four meta-analyses demonstrated that DOACs were consistently superior to VKAs in preventing recurrent stroke among patients with AF^[9,11,12,15]^. In particular, the network meta-analysis by Lopez-Lopez *et al* demonstrated significant reductions in recurrent stroke with apixaban (OR 0.79; 95% CI: 0.66–0.94) and high-dose dabigatran (OR 0.65; 95% CI: 0.52–0.81)^[16]^. These findings align with current clinical guidelines recommending DOACs over VKAs for most patients with non-valvular AF^[19–21]^. Nonetheless, warfarin remains a reasonable alternative for patients with stable therapeutic INR levels who tolerate the medication without adverse effects^[20]^.

One review demonstrated that adjusted-dose warfarin decreased stroke recurrence by approximately 40% compared to antiplatelet therapy, with an absolute risk reduction of 8.4% per year and a NNT of 12^[14]^. Regarding combination therapy, Ntaios *et al* reported that the addition of antiplatelet agents to oral anticoagulation did not improve stroke recurrence prevention and instead increased the risk of major bleeding (RR 1.45)^[13]^. These findings are consistent with recommendations from the Canadian Best Practice Guidelines and the American Heart and Stroke Association, which discourage dual therapy due to a lack of demonstrable benefit and increased bleeding risk^[19,20]^.

There remains debate regarding the optimal timing of OAC initiation after ischemic stroke. While American guidelines recommend initiation within 4–14 days post-stroke, the European Stroke Organization advocates for timing based on stroke severity^[19,21]^. However, evidence from Jiang *et al* suggests that early initiation is associated with a reduced risk of recurrent ischemic events (OR 0.68; 95% CI: 0.55–0.84), which challenges existing recommendations^[12]^.

Jiang *et al*^[12]^ conducted a meta-analysis comparing early versus delayed OAC initiation after ischemic stroke in AF patients and found that early initiation was associated with a reduced odds of recurrent ischemic events (OR 0.68; 95% CI 0.55–0.84). However, the analysis did not define specific time thresholds for “early” versus “delayed” initiation, nor stratify results by stroke severity. These limitations underscore the need for future studies with clearer timing windows and severity subgroup data.

In terms of safety, DOACs demonstrated a generally favorable bleeding profile relative to warfarin, with apixaban emerging as the agent with the lowest risk of major bleeding^[16]^. Rivaroxaban was less effective in reducing the risk of intracranial hemorrhage, while high-dose dabigatran was associated with an increased risk of gastrointestinal bleeding, particularly in elderly populations^[15]^. Although warfarin was found to increase bleeding risk in general populations, the findings from Murthy *et al* indicated no significant increase in bleeding when warfarin was reinitiated in AF patients following intracerebral hemorrhage (ICH), and it was associated with a reduction in thromboembolic events^[10]^. Study heterogeneity and selection biases, such as exclusion of patients at high risk for recurrent ICH, may explain these divergent results.

Economic evaluations generally support the cost-effectiveness of DOACs compared with warfarin for secondary prevention of cardioembolic stroke in AF, with apixaban often showing the most favorable cost–utility profile. U.S. Medicare models and secondary stroke prevention analyses have found that higher drug acquisition costs are offset by savings from reduced monitoring, fewer intracranial hemorrhages, and lower recurrence-related hospitalizations^[22,23]^. While cost-effectiveness varies by healthcare system, drug pricing, and adherence, these findings highlight the importance of considering both clinical and economic outcomes when selecting anticoagulants in resource-constrained settings.

This review identified several limitations in the current body of literature. A major limitation is the lack of specific focus on secondary stroke prevention in a subset of the included meta-analyses. Three reviews included populations with both primary and secondary stroke prevention needs, thereby limiting direct applicability of their conclusions to patients with prior stroke or TIA^[11,14,16]^. Future meta-analyses should stratify patient populations to enhance clinical relevance. Another methodological limitation is the overlap of primary studies across multiple meta-analyses. Redundancy, particularly in studies comparing DOACs to VKAs, introduces the risk of over-representation and potential bias. Such overlap can artificially inflate the weight of specific findings and compromise the accuracy of pooled estimates.

While the current evidence affirms the efficacy and safety of DOACs for secondary stroke prevention in AF, significant gaps remain. Subgroup analyses in frail older adults are scarce, as common eligibility criteria (e.g., recent bleeding, severe renal dysfunction, and need for intensive antiplatelet therapy) excluded many high-risk post-stroke patients. Observational data from the GARFIELD-AF registry indicate that elderly and frail AF patients have higher rates of both ischemic and bleeding events than younger cohorts, underscoring the importance of dedicated trials in this subgroup^[24]^. Evidence in advanced CKD (Chronic Kidney Disease) is largely observational; the scoping review by De Oliveira *et al*^[25]^ found no large randomized data for stage G5 CKD or dialysis, and dosing recommendations remain inconsistent. Patients with active cancer are rarely represented, despite elevated thrombotic and bleeding risk; a pooled analysis by Melloni *et al*^[26]^ found comparable stroke prevention efficacy of DOACs vs. VKAs in AF patients with cancer, but no data is available specific to prior-stroke populations. These gaps highlight the need for secondary-prevention trials that intentionally recruit underrepresented populations. Addressing these gaps has direct implications for clinical practice. For stroke neurologists, this means refining risk–benefit assessments when managing patients at the highest risk of recurrence or bleeding. For cardiologists, it reinforces DOAC preference while highlighting uncertainty in patients with comorbidities such as advanced CKD. For internists and primary care physicians, the key challenge remains sustaining adherence and monitoring comorbidities to ensure long-term safety. Together, these considerations support a more consistent and individualized approach across specialties.

Clinicians should adopt a patient-centered approach when selecting anticoagulant therapy, incorporating comorbidities, bleeding risk, renal function, and likelihood of adherence into decision-making. Further research is warranted to explore anticoagulant efficacy and safety in these understudied subgroups. Moreover, real-world observational studies and pragmatic clinical trials can help bridge the evidence gap left by randomized controlled trials. Despite these limitations, this umbrella review has notable strengths. It is the most recent and comprehensive synthesis of evidence regarding anticoagulant therapy for secondary prevention of cardioembolic stroke in AF. The inclusion of methodologically robust meta-analyses and the application of rigorous quality appraisal tools, such as AMSTAR-2, strengthen the validity of our conclusions.

## Conclusion

Anticoagulation strategies have evolved from traditional VKAs like warfarin to newer DOACs such as apixaban, rivaroxaban, and dabigatran. Evidence consistently supports the superiority of DOACs over warfarin in reducing stroke and systemic embolism risk, with apixaban demonstrating the most favorable safety and efficacy profile. Dabigatran, however, may pose similar risks of intracranial and gastrointestinal bleeding as warfarin. While early re-initiation of anticoagulation after ICH appears to reduce thromboembolic risk, optimal timing remains uncertain. Evidence remains limited for special populations such as the elderly, those with renal impairment, and post-ACS patients. An individualized, patient-centered approach to anticoagulant selection is essential, particularly in these high-risk groups.

## Data Availability

Data sharing is not applicable to this article, as no datasets were generated or analyzed during the current study.
